# Symptomatic Care in Multiple System Atrophy: State of the Art

**DOI:** 10.1007/s12311-022-01411-6

**Published:** 2022-05-17

**Authors:** Anna Grossauer, Victoria Sidoroff, Beatrice Heim, Klaus Seppi

**Affiliations:** Department of Neurology, Medical University of Innsbruck, Anichstrasse 35, A-6020 Innsbruck, Austria

**Keywords:** Multiple system atrophy, Symptomatic treatment, Motor and non-motor symptoms, Autonomic dysfunction

## Abstract

Without any disease-modifying treatment strategy for multiple system atrophy (MSA), the therapeutic management of MSA patients focuses on a multidisciplinary strategy of symptom control. In the present review, we will focus on state of the art treatment in MSA and additionally give a short overview about ongoing randomized controlled trials in this field.

## Introduction

Multiple system atrophy (MSA) is defined as a sporadic adult-onset neurodegenerative disorder characterized by a combination of various clinical features including autonomic dysfunction, parkinsonism, and ataxia with an inexorable progression of symptoms [1]. Depending on the predominant clinical presentation of motor symptoms, this neurodegenerative disorder can be classified into a parkinsonian subtype (MSA-P) and a cerebellar subtype (MSA-C) [2]. The estimated mean incidence rate is 0.6 cases per 100,000 persons per year in a US cohort, whereby the average annual incidence rate for ages 50 to 99 years is 3.0 cases per 100,000 persons per year [3]. The crude and age-adjusted prevalence rates are 3.3 respectively 4.4 cases per 100,000 persons per year [4]. MSA-P is more frequently observed than MSA-C in most countries [5–7], albeit, in Japan, the cerebellar phenotype is reported to be more common [8]. On average, disease onset is in the sixth decade of life and survival rates range from 6 to 10 years from the beginning of symptoms [9, 10].

Characteristic macroscopic changes in MSA include selective atrophy of the olivopontocerebellar and striatonigral system, which correlates with the two clinical phenotypes of motor features in MSA [11]. Furthermore, neurodegeneration is observed in the autonomic nervous system including for example the hypothalamus, Onuf’s nucleus, the intermediolateral column of the spinal cord and noradrenergic as well as serotoninergic brainstem nuclei [12]. Oligodendroglial cytoplasmic inclusions (GCIs), also called Papp-Lantos bodies, contain abundant misfolded α-synuclein aggregates and are the histopathological hallmark of MSA [13–15].

To date, there are no approved disease-modifying therapies for MSA. However, a broad spectrum of research approaches is currently in development for clinical trials, which could yield new opportunities in the treatment of this relentlessly progressive neurodegenerative disorder. So far, the therapeutic management of MSA patients focuses on a multidisciplinary strategy of symptom control with the patient and quality of life at the center of interest. In the present review, we will focus on state of the art, symptomatic treatment in multiple system atrophy and will additionally give a short overview about ongoing randomized controlled trials (RCTs) in this field.

## Symptomatic Treatment

Most therapeutic approaches in MSA patients are prescribed off-label and based on results from studies investigating other disorders or from uncontrolled studies, retrospective analyses, case reports, or expert opinion. Albeit there is only a limited number of controlled clinical trials pharmacological and non-pharmacological treatment strategies and the involvement of a multidisciplinary team may have beneficial effects on quality of life in MSA patients. Tables 1 and 2 provide information about possible treatment options in MSA.

**Table 1:** Treatment options for motor symptoms and sleep problems in MSA

Symptoms	Pharmacological*	Non-pharmacological	Comments
*Motor symptoms*			
Parkinsonism	• Levodopa/DCC (up to 1000 mg/day) • Amantadine (100–400 mg/day) • Anticholinergics	• Physiotherapy • Occupational therapy • Speech therapy	• As dopamine agonists show poor efficacy and may involve severe side effects, particularly worsening of OH, they should not be considered a therapeutic option • If dystonia is a prominent feature, anticholinergics may be considered
Cerebellar ataxia	-	• Physiotherapy • Occupational therapy • Speech therapy • rTMS	• Some beneficial effects have been reported with the off-label use of propanolol, baclofen, amantadine, gabapentin or buspirone • Daytime sedating drugs or muscle relaxants (e.g., benzodiazepines or baclofen) can have worsening effects on balance and increase the risk of fall • Consider injury protective garments in case of regular falling
Dystonia	• Amantadine • Anticholinergics • BoNT-A	• Physiotherapy	• Risk of dry mouth, urinary retention, or cognitive dysfunction with anticholinergics • Possible exacerbation of disease-associated dysphagia with the treatment of cervical dystonia with BoNT-A
Dysphagia and dysarthria	-	• Swallowing and speech therapy	• Dietary adjustments (use of thickeners for fluid, adopt meal size, and consistency) • In severe cases with aspiration, consider gastrostomy tube feeding
Drooling	• Oral (e.g. glycopyrrolate) or topic anticholinergics • BoNT-A (salivary glands)	• Swallowing and speech therapy	• Risk of dry mouth, urinary retention, or cognitive dysfunction with anticholinergics • Risk of dry mouth or aggravation of dysphagia with BoNT-A
*Sleep problems*			
REM sleep behavior disorder	• Clonazepam (0.5–2 mg at bedtime) • Melatonin (2–5 mg at bedtime)	• Safe sleeping environment for both the patient and the bed partner for injury prevention (e.g. placing a mattress on the floor, padding corners of furniture, or removing potentially dangerous objects from the bedroom)	• Review of the current drug regimen and consider removal or dose reduction of aggravating drugs (e.g. TCAs and SSRIs) • In refractory patients treatment with cannabidiol (50–300 mg ad bedtime), gabapentin (300–800 mg at bedtime), pregabalin (25–100 mg at bedtime) or sodium oxybate (4.5–9 g/day) can be considered
Nocturnal stridor	-	• NPPV or CPAP • Tracheostomy	• Tracheostomy should be considered in the advanced disease stage for severe stridor and in case of stridor during wakefulness with immobile vocal cords on laryngoscope

*CPAP*, continuous positive airway pressure; *BoNT-A*, botulinum toxin A; *l-DOPS*, l-threo-dihydroxyphenylserine (l-threo-DOPS, droxidopa); *NPPV*, non-invasive positive pressure ventilation; *SSRI*, selective serotonine reuptake inhibitors; *SNRI*, 5HT-norepinephrine reuptake inhibitor; *REM*, rapid eye movement

^*^Some of the drugs listed for a specific indication are not approved for this indication. Such off‐label use for a specific indication is reasonable when this would benefit the individual patient, but such off‐label use is not without its dangers

**Table 2:** Pragmatic management of autonomic dysfunction in MSA

Orthostatic hypotension	
Review current drug regimen	Consider removal or dose reduction of aggravating drugs (e.g. atypical antipsychotics, tricyclic antidepressants, antagonists of α-adrenoceptors to treat prostatism, antihypertensive drugs such as diuretics or calcium channel blockers)
Non-pharmacological treatment	• Sleeping in head-up position • Fragmentation of meals • Physical counter manoeuvres such as squatting, bending over forward or leg crossing with tension of the thigh, bottom and calf muscles (party position) at the onset of pre-syncopal symptoms • Avoidance of low-sodium and carbohydrate rich meals • Increased water (2–2.5 l/day) and salt intake (> 8 g or 150 mmol /day) • Avoid hot beverages and alcohol • Elastic stockings
Pharmacological treatment*	• *Expansion of intravascular volume:Fludrocortisone 0.05–0.2 mg/day * • *Increase of peripheral vascular resistance:Midodrine 2.5–30 mg/day* Droxidopa 100–600 mg × 3/day Atomoxetine (10–36 mg/day) • *Other treatment options*: Domperidone, Ephedrine, Etilefrine, Pyridostigmine Octreotide (s.c. before meals) may be helpful for postprandial hypotension
Supine hypotension	
Non-pharmacological treatment	• Sleep with the head of the bed up 6 to 9 inches • Rest on a semirecumbent chair with feet on floor during the day • Encourage snack with high-carbohydrate content before bedtime • Reduce fluid intake at bedtime • Avoid evening doses of pressor agents
Pharmacological treatment*	• Use of short-acting antihypertensive drugs (e.g. nitrates such as transdermal nitroglycerin overnight, losartan, short-acting calcium channel blocker such as nifedipine, minoxidil, hydralazine, or clonidine early in the evening)
Urogenital symptoms	
Review current drug regimen	Consider removal or dose reduction of aggravating drugs (e.g.: polyuria due to diuretics, incontinence due to antagonists of α-adrenoceptors to treat prostatism or arterial hypertension, urinary retention due to anticholinergics including tricyclic antidepressants or agonists of ß-adrenoceptors or midodrine)
Non-pharmacological treatment	• Noctural polyuria: Reduced fluid intake or avoiding coffee in the evening • Urge incontinence: Check for bladder infections, antibiotic treatment if positive, Bladder training, Tibial nerve neuromodulation • Incomplete bladder emptying: Clean intermittent self-catheterization if postmicturitional residual volume is > 100 ml in several measurements, long-term urethral catheter (permanent urethral or suprapubic catheterization) when neurogenic bladder symptoms become unmanageable • Erectile dysfunction: Intraurethral suppositories, intraurethral or intracavernosal administration of vasoactive drugs, vacuum-assisted erectile devices, external prosthetic devices, low-intensity shock wave therapy
Pharmacological treatment*	• *Noctural polyuria:* Desmopressin spray (10–40mcg/night) Consider development of hyponatremia • *Urge incontinence:* Mirabegron 25 – 50 mg /day Anticholinergics: Trospiumchloride 20–40 mg/day or Solifenacin 5–10 mg/day Consider increased risk of worsening urinary retention with anticholinergics and mirabegron Intravesical botulinum toxin • *Incomplete bladder emptying:* a1-adrenergic blockers: Tamsulosin 0.4 mg/day; Prazosin 1–3 mg/day; Moxisylyte 10–30 mg/day Consider risk of worsening of OH • *Erectile dysfunction:* Oral PDE-5 inhibitor: 50 mg Sildenafil, 10 mg Vardenafil, 20 mg Tadalafil Consider risk of worsening of OH
Constipation	
Non-pharmacological treatment	• Consider removal of aggravating drugs (e.g., opioids or anticholinergics) • Increased exercise • Sufficient or additional fluid intake • Use of stool softener • Probiotics, prebiotic fibre, fibre (e.g., bran, unprocessed foods, and fibre additives) or oral fiber supplements (such as psyllium or methylcellulose) • Enemas and manual disimpaction may be required in severely affected patients
Pharmacological treatment*	• Lactulose (20–60 g/day) and laxatives such us sodiumpicosulfate (5–10 mg/day) or macrogol (polyethylene glycol 13–39 g/day) • Prokinetic drugs (e.g. prucalopride) • Secretagogues (e.g. lubiprostone, linaclotide, or plecanatide) • Botulinum toxin A injections into the puborectalis for muscle outlet-obstruction constipation

*OH*, orthostatic hypotension

^*^Some of the drugs listed for a specific indication are not approved for this indication. Such off‐label use for a specific indication is reasonable when this would benefit the individual patient, but such off‐label use is not without its dangers

## Management of Motor Symptoms

### Pharmacological Treatment

#### Parkinsonism

Parkinsonian symptoms are observed in nearly 90% of MSA cases and they include rigidity, postural instability, tremor, and freezing of gait besides the core feature of bradykinesia [6]. Treatment of parkinsonism targets the dopaminergic system and comprises the use of levodopa administered in combination with a peripheral decarboxylase inhibitor such as carbidopa or benserazide. The use of levodopa relies on a broad clinical experience; however, there is only low evidence for its therapeutic effect in MSA from clinical trials [1]. Even if a poor or lack of levodopa response is a diagnostic criterion for MSA [16], reports of initial therapeutic response rates range from 30 to 70% [17–19], but are accompanied by the evolvement of motor complications in a substantial number of these patients including wearing-off fluctuation in 23%, off-dystonia in 20%, on–off fluctuations in 14%, and peak dose dyskinesias in 11% [10]. Beneficial response rates to levodopa therapy are more frequently observed in MSA-P patients compared to MSA-C patients [10]. According to consensus criteria [16], unresponsiveness to levodopa should only be regarded after a treatment period of at least 3 months in daily doses of up to 1 g without any significant clinical improvement of parkinsonian symptoms. Levodopa response may be considered positive by improvement of 30% or more on the motor examination part of the Unified Parkinson’s Disease Rating Scale (UPDRS) part III or on part II of the Unified Multiple System Atrophy Rating Scale (UMSARS) [16]. Intriguingly, the withdrawal of levodopa occasionally causes worsening of parkinsonism in MSA patients without apparent response on levodopa therapy [1]. On the other hand, levodopa-induced dyskinesia, predominantly of dystonic nature and typically involving the craniocervical regions, may emerge, already after short-term use, in MSA-P patients responsive to levodopa [20].

Dopamine agonists showed beneficial effects only in a small proportion of MSA patients [21] and as this drug class is also associated with increased side effects compared to levodopa, particularly worsening of OH, it should not be considered first-line therapy for parkinsonism in MSA [1].

The MAO-B inhibitor rasagiline was investigated as another treatment option in MSA-P patients but failed to show significance over placebo in a large RCT [22]. A small placebo-controlled trial demonstrated a tendency towards an improvement in UPDRS III scores with amantadine, which is thought to work as an *N*‑methyl-d‑aspartate receptor antagonist [23]. Despite the low evidence for the efficacy of amantadine in the symptomatic treatment of parkinsonism in MSA patients, it may be considered an alternative or additional option [1]. Amantadine has a low side effect profile causing sometimes peripheral edema, dizziness, and psychotic features such as delusions or hallucinations. On the other hand, no meaningful treatment effect for parkinsonian symptoms in MSA could be found in a small placebo-controlled cross-over trial with the anti-glutamatergic agent riluzole 100 mg twice daily, albeit the small sample number of this study was mentioned as a limiting aspect [24]. Moreover, a subsequent large trial also failed to demonstrate a significant effect of riluzole on survival and disease progression in Parkinson plus disorders, including Progressive Supranuclear Palsy (PSP) and MSA patients [25], thus making evidence for the use of riluzole in MSA vanishing small.

#### Cerebellar Ataxia

Currently, there are no pharmacologic treatment options available for cerebellar ataxia in MSA, which provide an evidence-based improvement of ataxic symptoms from large, controlled clinical trials, although some beneficial effects with the off-label use of propanolol, baclofen, or amantadine have been described [26] as well as with the administration of gabapentin [27] or the off-label prescription of buspirone for upper limb ataxia [28]. However, attention has to be drawn to the fact that daytime sedating drugs or muscle relaxants (e.g., benzodiazepines or baclofen) can have worsening effects on balance and increase the risk of falls.

#### Motor Impairment

In addition to the pharmacological strategies specifically targeting parkinsonism or cerebellar ataxia in MSA, selective serotonin reuptake inhibitors (SSRIs) have been investigated for their efficacy to improve motor symptoms in MSA. Encouraging results on motor impairment have been reported for paroxetine compared to placebo in a small placebo-controlled, randomized study with trends towards a greater reduction in UPDRS III scores in patients treated with paroxetine compared to placebo and a significant improvement in the sub-categories “Speech”, “Finger Taps,” and “Alternating Movements of the Hands” [29]. A more recent RCT studied the use of fluoxetine in MSA patients and failed to demonstrate superiority of this compound over placebo on the total UMSARS score; however, improvements in motor symptoms as assessed by the UMSARS part II score and in emotional secondary outcomes have been shown [30]. Intriguingly, SSRIs may also have a disease-modifying effect in MSA by increasing levels of glial-derived neurotrophic factor (GDNF) and brain-derived neurotrophic factor (BDNF) [31], which represent neurotrophic factors playing an important role in neuroprotection, and by reducing inflammation-mediated neurodegenerative processes [32].

#### Dystonia

The reported prevalence for focal dystonia in MSA, such as anterocollis or blepharospasm, is between 12 and 46%, thus making it a common feature in this neurodegenerative disorder [20, 33]. Even though there is no RCT available for the symptomatic treatment of dystonia in MSA, there is some evidence for a favorable effect of botulinum toxin type A injections in the treatment of dystonia in atypical Parkinsonian disorders [34]. In cervical dystonia, especially anterocollis, botulinum toxin injections should be used with caution due to a possible exacerbation of disease-associated dysphagia [35]. In the absence of any RCTs with other drugs, off-label use of anticholinergics or amantadine has been reported to relief symptoms of dystonia in some MSA patients [26]. One has to consider, however, that anticholinergic drugs can cause central adverse effects such as cognitive dysfunction, behavioural disturbances or confusion, as well as peripheral adverse effects as xerophthalmia and xerostomia, blurred vision, urinary retention, and constipation.

#### Dysarthria, Dysphagia, and Drooling

To date, there are no approved medical therapies for the symptomatic treatment of dysarthria, dysphagia, or aspiration risk in MSA. Patients may benefit from early support by a speech-language pathologist, which are of utmost importance in the multidisciplinary care of MSA patients [36]. Food modifications like slower eating, soft solid diet, and even placing a percutaneous endoscopic gastrostomy (PEG) may be considered, if dysphagia gets worse. Anticholinergic drugs may be an option to reduce drooling in MSA patients [37] but have to be used cautiously due to undesirable side effects such as cognitive impairment or dry mouth. Botulinum toxin A injections into the salivary glands were studied as a treatment opportunity for drooling as well and yielded promising results in addition to a good safety profile [38–40], though rarely occurring aggravations of swallowing problems should be considered with its administration [35].

### Non-pharmacological Treatment Approaches

Deep brain stimulation of the subthalamic nucleus is nowadays an established evidence-based method for the treatment of motor fluctuations and dyskinesia in advanced Parkinson’s disease [41], but failed to show a sustained benefit in MSA patients and is therefore currently not recommended as a non-pharmacological treatment approach for motor symptoms in MSA [42–44].

Repetitive transcranial magnetic stimulation (rTMS) is a noninvasive technique to deliver repeated magnetic pulses over the scalp, which generates a relatively focused electromagnetic field that can modulate cortical neuronal excitability [45], and has been studied with sham-controlled randomized trials for the treatment of motor and non-motor symptoms in PD [46, 47]. More recently, preliminary research from small-sized short term randomized sham-controlled trials has suggested improvements of motor symptoms in MSA with rTMS over the primary motor cortex [48] and of the cerebellum [49]. The results of these two trials [48, 49] provide interesting insights for the use of rTMS as symptomatic treatment option in MSA.

As the effect of symptomatic pharmacological therapy is mostly limited, exercise and medical rehabilitation play important roles in improving symptoms and patients’ quality of life, even though strong evidence from RCTs is limited [1]. Nevertheless, a randomized-controlled trial of patients with mild to moderate MSA obtaining occupational therapy showed significant improvement of motor function and activities of daily life [50]. Furthermore, an improvement in health-related quality of life was associated with the receipt of occupational therapy supporting importance of non-pharmacological treatment approaches in neurodegenerative disorders like MSA with versatile impacts on everyday life of patients.

Evidence from open label trials and case reports suggests that parkinsonism as predominant motor feature in MSA may benefit from physiotherapy [1, 51–53] as coordination, balance, and gait impairment may be improved with intensive physiotherapy as well as with resistance training and challenge-oriented gait and balance training in degenerative cerebellar disorders [54–57]. In case of regular falling injury, protective garments should be considered.

## Management of Autonomic Dysfunction

Early onset, generalized and rapidly progressive autonomic failure is typical of MSA. Rates of autonomic failure of up to 60% are reported at the disease onset [1].

### Orthostatic Hypotension

Patients with neurogenic OH (nOH) often suffer from orthostatic dizziness and hypotension leading to syncopes, fatigue, weakness, or coat hanger pain. While OH affect about 75% of MSA patients [21, 58], symptoms of OH may even predate motor onset of the disease. Longitudinal studies of patients with isolated autonomic failure, indeed, found that 24 to 34% of them phenoconverted after 4 years and more to an α-synucleinopathy, with 24 to 63% of them developing MSA [59–61]. Pathophysiological factors include noradrenergic cardiac and extracardiac denervation, as well as the lack of arterial baroreflexes that are often seen in α-synucleinopathies [62].

Respecting the fact that normalization of standing blood pressure in MSA patients is a very difficult process and even sometimes not realizable, nOH treatment is primarily focusing on reduction of symptom burden and improvement of quality of life. Generally, a combination of pharmacological and non-pharmacological interventions might provide best symptom control of nOH. The first step in the treatment of symptomatic OH is however a rigorous review of the current drug regimen with discontinuing or reducing the dose of aggravating drugs such as atypical antipsychotics, tricyclic antidepressants, antagonists of α-adrenoceptors to treat prostatism, antihypertensive drugs such as diuretics or calcium channel blockers, and sometimes even antiparkinsonian dopaminergic drugs (dopamine agonists first) based on an individual risk–benefit assessment.

Non-pharmacological treatment options include the use of custom-fitted compression stockings or elastic abdominal binders, raising the head of the bed by 20–45° when sleeping to increase intravasal volume and reduce hypotension in the morning, but also lifestyle changes such as an adequate intake of fluid and salt, avoiding exposure to hot, humid environments and performing physical activity programs with recumbent or sitting exercises [1, 63]. Because of promising results of elastic abdominal binders for OH treatment in PD patients [64] a RCT is currently investigating MSA and PD patients with OH (NCT04920552).

Pharmacologic approaches for the treatment of nOH in MSA include two complementary strategies, which aim to expand intravascular volume with the mineralocorticoid agonist fludrocortisone and to increase peripheral vascular resistance with pressor agents like the α-adrenergic agonist midodrine, the norepinephrine precursor droxidopa, or the reuptake inhibitor atomoxetine.

Midodrine has been proven to be effective for patients with nOH including MSA patients [65, 66]. By enhancing sympathetic activity, a double-blind, randomized, crossover study demonstrated that a single-dose pyridostigmine also significantly improves standing blood pressure in patients with OH without worsening supine hypertension [67].

Three RCTs enrolling over 400 patients with nOH lead to FDA approval of droxidopa in the US [68–70]. Two of these trials included MSA patients with a total number of 95 participants and positive effects on OH symptoms have been reported for patients with nOH [68, 70]. Comparable symptomatic and safety results for the treatment of nOH have been reported for atomoxetine compared to midodrine [71, 72]. However, although a phase II randomized-controlled trial with ampreloxetine, another norepinephrine reuptake inhibitor, for the treatment of neurogenic OH suggested improvement of orthostatic symptoms in a phase II trial [73], the primary endpoint of two phase III trials [74, 75] was not achieved for ampreloxetine for the treatment of nOH. A pre-specified subgroup analysis by disease type, however, suggests a benefit in patients with MSA receiving ampreloxetine (NCT03829657) [75].

### Supine Hypertension

NOH in MSA typically coexist with supine hypertension and pharmacologic treatment of one usually exacerbates the other. The treatment of supine hypertension in MSA aims minimization of the risk of end organ damage without worsening symptoms of nOH [76]. Treatment options to manage supine hypertension in MSA include non-pharmacological measures such as avoidance of the supine position during daytime and raising the head of the bed at night as well as the careful use of short-acting antihypertensive drugs, that can cause aggravation of nOH. Therefore, patients should be warned about this side effect when they have to get up at night and recommend using a urinal or bedside commode to avoid falls [77].

### Urogenital Dysfunction

Urinary dysfunction is a common symptom affecting most of the MSA patients during the disease course [78–80] and includes urinary urgency with incontinence (overactive bladder), urinary retention (detrusor underactivity), or a combination of both [80]. To date, there are no placebo-controlled trials for pharmacotherapy of urinary dysfunction in MSA.

Primarily, bladder dysfunction management in MSA should include a review of the current drug regimen for possible aggravated bladder dysfunction. This may include polyuria due to diuretics, incontinence due to antagonists of α-adrenoceptors to treat prostatism or arterial hypertension, and urinary retention due to anticholinergics like tricyclic antidepressants or agonists of ß-adrenoceptors. Urge incontinence caused by detrusor hyperreflexia and sphincter detrusor dys-synergy may be alleviated by applying anticholinergic substances or the selective β3-adrenergic receptor agonist mirabegron [63]. Antimuscarinic drugs can cause xerophthalmia and xerostomia and aggravate urinary retention and constipation, while mirabegron is devoid of anticholinergic side effects but with urinary retention, abdominal pain, and hypertension as potential side effects. Cognitive deterioration can occur with antimuscarinic agents especially if there is relevant CNS penetrance such as with oxybutynin or tolterodine and is less likely with those with minimal CNS penetrance such as solifenacin and trospium chloride [81]. Single case studies reported improved nocturia with intranasal desmopressin [82], but development of hyponatremia is common. Alternative treatment modalities for overactive bladder include tibial nerve neuromodulation or intravesical botulinum toxin [83–85].

Neurogenic incomplete bladder emptying can sometimes improve with urethra-oriented alpha-adrenergic blockers (tamsulosin, prazosin, or moxisylyte), but OH can be aggravated [1]. To avoid consecutive urinary tract infections, in patients with post-void residual volumes above 100 ml usage of intermittent self-catheterization, if patient or a caregiver can perform this after education, or an indwelling catheter, usually suprapubic, may be required [63, 86, 87]

For the treatment of erectile dysfunction, sildenafil has been proven to be efficacious in a small RCT in MSA [88] such as phosphodiesterase-5 inhibitors can be recommended as first-line treatment strategy for erectile dysfunction in male patients with MSA. All phosphodiesterase-5 inhibitors should be used cautiously in MSA patients, because they can induce systemic vasodilation that can cause dramatic reductions in blood pressure [47]. Urological consultation should be done for second line options which might include intraurethral suppositories, intraurethral or intracavernosal administration of vasoactive drugs, vacuum-assisted erectile devices, external prosthetic devices, or low-intensity shock wave therapy, alone or combined with a PDE-5 inhibitor [89]. For the treatment of female sexual dysfunction, use of vaginal lubricators and hormone therapy may be considered [63].

### Constipation

General measures for the alleviation of constipation in MSA include increased exercise, removal of aggravating drugs (e.g., opioids or anticholinergics), sufficient fluid intake, high-fiber diet (e.g., bran, unprocessed foods, and fiber additives) and the use of stool softeners before oral fiber supplements (such as psyllium or methylcellulose) or polyethylene glycol can be tried to improve constipation [36, 63, 90]. In refractory patients, it is worth introducing empirically pro-kinetic drugs (e.g. prucalopride) or secretagogues drugs (e.g. lubiprostone, linaclotide, or plecanatide), all of them causing diarrhea as most common side effect [47, 91]. Enemas and manual disimpaction may be required in severely affected patients.

## Management of Sleep Problems

Sleep problems in MSA are almost universal [2] and are due to a multitude of factors including primary dysfunction of sleep–wake-cycle regulation, REM sleep behavior disorder (RBD), and secondary effects of motor and non-motor symptoms on sleep onset and maintenance such as immobility, depression, or nocturia. In addition, effects of medications on sleep, as well as the impact of co-morbid conditions like sleep-disordered breathing or stridor, may affect sleep. Therapeutic decisions for sleep problems are pragmatic without formal evidence from RCTs. Careful history taking—often including information from a spouse or care giver—is essential to identify the most likely and relevant underlying causes. It is worth considering polysomnographic verification. A first step should always be a careful review of the patient’s drug history with an aim to identify drug-induced insomnia, which can occur following the introduction of many drugs (e.g. α-blockers, β-blockers, antidepressants, angiotensin-II receptor blockers) including parkinsonian drugs [92]. Treatment options include optimizing therapies for parkinsonian features to improve nocturnal symptom control, treatment of non-motor symptoms like nocturia or depression, counseling about sleep hygiene as well as the addition of drugs promoting sleep or wakefulness (e.g. Z-drugs such as eszopiclone, zolpidem, zaleplon; benzodiazepines such as triazolam, temazepam; sleep promoting antidepressants such as trazodone, low-dose doxepin, or suvorexant) [92].

RBD affects almost all MSA patients. General measures for the treatment of RBD include a careful review of the current drug regimen for potential aggravators (e.g. TCAs and SSRIs) [93] and recommendation for maintenance of a safe sleeping environment for both the patient and the bed partner for injury prevention (e.g. placing a mattress on the floor, padding corners of furniture, or removing potentially dangerous objects from the bedroom). Because there are no controlled trials of any medication used to treat RBD in MSA available, management relies on recommendations for RBD in general. Clonazepam and Melatonin given as a single dose before bed-time are considered first-line treatment for RBD in PD [94], although the former may aggravate nocturnal stridor, sleep apnea, or daytime ataxia. Melatonin can be combined with Clonazepam and should be the first line choice in case clonazepam is not efficient or contraindicated [1, 95]. Other treatment options for the treatment of RBD may include gabapentin or pregabalin at bedtime, cannabidiol at bedtime or sodium oxybate [1].

For sleep disordered breathing like obstructive sleep apnea, positive airway pressure (CPAP) or bi-level positive airway pressure provides benefit [36, 96, 97]. Small studies support the use of home non-invasive positive pressure ventilation (NPPV) and continuous positive airway pressure (CPAP) ventilation for nocturnal stridor [96, 97] which is a frequent symptom in MSA associated with respiratory failure and sudden death during sleep [1, 98]. These kinds of non-invasive home ventilation are usually well tolerated, especially early in the disease [99]. Tracheostomy can be considered in the advanced disease stage for severe stridor and in case of stridor during wakefulness with immobile vocal cords on laryngoscope [100].

## Ongoing RCTs for Symptomatic Treatment in MSA

All completed RCTs that have included MSA patients for symptomatic treatment are summarized in Table 3. Currently, there are several clinical trials going on investigating treatment options for autonomic and gait symptoms, motor function in general, parkinsonism, cerebellar ataxia, orthostatic hypotension, and supine hypertension in MSA (Table 4). First results for treatment with safinamide in MSA were recently published on clinicaltrials.gov (NCT03753763). No significant change in motor performance following treatment with safinamide was reported.

**Table 3:** Randomized controlled trials assessing symptomatic therapies in MSA

Intervention	n (MSA)	Trial	Target	Primary outcome	Results	Author	Comments
Droxidopa	181 (51)	RCT Phase III	OH	OHSA item 1	-	Biaggioni 2015	Mixed population ; secondary endpoints (total OHQ, QoL) fulfilled
Droxidopa	162 (44)	RCT Phase III	OH	OHQ total score	+	Kaufmann 2014	Mixed Population; positive impact on standing BP
Midodrine	171 (15)	RCT Phase III	OH	Standing systolic BP, OH symptoms, global symptom relief score	+	Low 1997	Mixed population
Midodrine	97 (27)	RCT Phase III	OH	1 hour standing BP	+	Jankovic 1993	Mixed population
Pyridostigmine (+/- Midodrine)	58 (17)	RCT Phase II	OH	Standing diastolic BP	+	Singer 2006	Mixed population, single dosing
Atomoxetine vs. Midodrine	50 (8)	OL Phase II	OH	Orthostatic BP	+	Byun 2020	Mixed population, symptomatic improvement only with atomoxetine
Atomoxetine vs. Midodrine	69 (21)	Single blind Phase II	OH	Upright systolic BP after 1 minute	+	Ramirez 2014	Mixed population
Ampreloxetine	34 (18)	RCT Phase II + OLE	OH	Seated systolic BP, OHSA item 1	+	Kaufmann 2021	Mixed population
Ampreloxetine	N.A.	RCT Phase III	OH	OHSA item 1 & PGI-S	-	not published yet	
Rasagiline	174 (174)	RCT Phase II	Motor sym.	Total UMSARS score	-	Poewe 2015	
Amantadine	8 (8)	RCT Phase II	Motor sym.	N.A.	N.A.	Wenning 2005	Exploratory study design with a small sample number; trend towards reduction of UPDRS-III score
Riluzole	10 (10)	RCT Phase II	Motor sym.	N.A.	N.A.	Seppi 2006	Exploratory study design
Riluzole	767 (404)	RCT Phase II	Motor sym., ADL, survival	Survival	-	Bensimon 2009	Mixed population with PSP and MSA patients
Paroxetine	20 (20)	RCT Phase II	Motor sym.	N.A.	N.A.	Friess 2006	Exploratory study design; significant improvement in motor abilities of the upper limbs and speech
Fluoxetine	81 (81)	RCT Phase II	Motor sym., QoL	Total UMSARS score	-	Rascol 2021	Improvement in UMSARS-II and MSA-QoL
rTMS	15 (15)	RCT	Motor sym.	UMSARS-II score	+	Wang 2016	
rTMS	50 (50)	RCT	Ataxia	N.A.	N.A.	Song 2020	Exploratory study design with functional and clinical endpoints; significant decrease in SARA score
Occupational therapy	17 (17)	RCT	Motor sym. (ADL), QoL	N.A.	N.A.	Jain 2004	Exploratory study design; significant reduction of total UPDRS and PDQ-39 scores
Botulinum toxin A	20 (6)	RCT	Siallorhea	Drooling	+	Mancini 2003	Mixed population

All but the Atomoxetine vs. Midodrine trial [71] are placebo-controlled trials or trials with sham-intervention (rTMS trials) or no intervention (occupational therapy)

*RCT* Randomized controlled trial, *OLE* Open-label extension, *OH* Orthostatic hypotension, *ADL* Activities of daily living, *QoL* Quality of Life, *rTMS* Repetitive transcranial magnetic stimulation, *OHSA* OH-symptom assessment, *OHQ* OH-questionnaire, *BP* blood pressure, *UMSARS* Unified multiple system atrophy rating scale, *UPDRS* Unified Parkinson’s disease rating scale, *SARA* Scale for the assessment and rating of ataxia, *N.A.* not applicable

* + *primary endpoint positive, - primary endpoint negative

**Table 4:** Ongoing RCTs for symptomatic treatment in MSA

Symptom	Therapy	Phase	Clinicaltrials.gov ID
*Autonomic and gait symptoms*	PPN-DBS	Pilot study	NCT03593512
*Motor function*	Physiotherapy STN-DBS and Spinal Cord Stimulation	II Pilot study	NCT04608604 NCT04617873
*Cerebellar Ataxia*	Tllsh2910 Transcranial direct current stimulation	III Pilot study	NCT03901638 NCT04092556
*Orthostatic hypotension*	Midodrine and droxidopa Droxidopa Automated abdominal binder Abdominal binder vs. Midodrine Abdominal binders	I IV I/II I NA	NCT02897063 NCT02586623 NCT03482297 NCT04620382 NCT04920552
*Supine hypertension*	CPAP	NA	NCT03312556

*PPN-DBS*, pedunculopontine nucleus deep brain stimulation; *STN-DBS*, subthalamic nucleus deep brain stimulation; *CPAP*, continuous positive airway pressure

## Conclusion

MSA is a devastating neurodegenerative disease with a broad spectrum of symptoms. Due to the multifaceted presentation of its clinical phenotype and progressive course of MSA as a multisystem disorder, its management is usually complex and should involve a multidisciplinary team consisting of neurologists along with specialists from other disciplines including neuro-urology, cardiology, sleep medicine, or rehabilitation and other allied healthcare partners as well as palliative care [1, 36]. Furthermore, social services are critical to support MSA patients and their relatives. The neurological team represents the coordinating central contact point for MSA patients and their relatives in this setting.

Although therapeutic symptomatic decisions in MSA are in most instances pragmatic without formal evidence from RCTs, there are symptomatic treatment options for motor symptoms, autonomic failure, and sleep disturbances available [1]. Tables 1 and 2 give an overview of these treatment options. Sometimes forgotten but of central importance are non-pharmacological treatments starting from simple tips to avoid OH symptoms (including lifestyle changes, compression stockings, or head up tilt while sleeping), to regular therapies including exercise and medical rehabilitation to keep the current mobile state and avoid falls. Empirically, patients should be provided access to rehabilitation programs regularly, e.g. at least once a year. Also, the treatment of depression and pain in MSA, other common symptoms in MSA, is based on pragmatic recommendations without firm evidence for efficacy from controlled clinical trials. SSRIs are the drug of choice for managing depressive symptoms in MSA and fluoxetine, indeed, ameliorated emotional and social quality of life in a placebo-controlled trial of patients with MSA [30, 101]. Botulinum toxin injections may be helpful for pain associated with dystonia, while other types of pain may benefit from duloxetine, gabapentin, or pregabalin. Moreover, there is anecdotal evidence that medical cannabis [102] or cannabidiol might be beneficial for the treatment of pain [103].

Although there is a lack of formal evidence from RCTs, palliative and supportive care should be discussed early in the disease, as in other progressive neurological disorders [104, 105]. Intriguingly, a RCT of patients with progressive neurological disorders including MSA reported that short-term integrated palliative care was associated with trends toward reduced healthcare costs and reduced symptom burden, although there was no impact on survival [106].

If possible, MSA patients should be followed up in a center of competence which should have access to the most common treatment strategies and options including access to ongoing trials running on-site or elsewhere.

Pharmacological treatment must be used wisely as many medications implement worsening of other symptoms like OH. Therapeutic decisions are in most instances pragmatic without formal evidence from RCTs. To generate treatment relevant data and keeping in mind that MSA is a rare disease, there should be a bigger focus on international and multicentric studies with comparable outcome parameters including quality of life.

However due to the nature of the disease, there is still an unmet need of disease-modifying therapy for MSA.
